# Effect of Low Temperature and Wheat Winter-Hardiness on Survival of *Puccinia striiformis* f. sp. *tritici* under Controlled Conditions

**DOI:** 10.1371/journal.pone.0130691

**Published:** 2015-06-17

**Authors:** Lijie Ma, Jiaxing Qiao, Xinyu Kong, Yiping Zou, Xiangming Xu, Xianming Chen, Xiaoping Hu

**Affiliations:** 1 State Key Laboratory of Crop Stress Biology for Arid Areas, College of Plant Protection, Northwest A&F University, Taicheng Road 3, Yangling 712100, China; 2 East Malling Research, New Road, East Malling, ME19 6BJ, Kent, United Kingdom; 3 Agricultural Research Service, United States Department of Agriculture and Department of Plant Pathology, Washington State University, Pullman, WA 99164-6430, United States of America; Louisiana State University Agricultural Center, UNITED STATES

## Abstract

Wheat stripe rust, caused by *Puccinia striiformis* f. sp. *tritici* (*Pst*), is one of the most important diseases of wheat worldwide. Understanding the survival of *Pst* during the overwintering period is critical for predicting *Pst* epidemics in the spring. Real-time quantitative PCR (qPCR) methods quantifying *Pst* DNA and RNA (cDNA) were developed and compared for the ability to quantify viable *Pst* in leaf tissues. Both qPCR of DNA and RNA can provide reliable measurement of viable *Pst* in plant tissues prior to the late sporulation stage for which qPCR of DNA gave a much higher estimate of fungal biomass than qPCR of RNA. The percentage of *Pst* biomass that was viable in detached and attached leaves under low temperatures decreased over time. *Pst* survived longer on attached leaves than on detached leaves. The survival of *Pst* in cultivars with strong winter-hardiness at 0°C and -5°C was greater than those with weak winter-hardiness. However, such differences in *Pst* survival among cultivars were negligible at -10, -15 and -20°C. Results indicated that *Pst* mycelia inside green leaves can also be killed by low temperatures rather than through death of green leaves under low temperatures. The relationship of *Pst* survival in attached leaves with temperature and winter-hardiness was well described by logistic models. Further field evaluation is necessary to assess whether inclusion of other factors such as moisture and snow cover could improve the model performance in predicting *Pst* overwintering potential, and hence the epidemic in spring.

## Introduction

Wheat stripe rust, caused by *Puccinia striiformis* Westend. f. sp. *tritici* Erikss. (*Pst*), is one of the most important diseases of wheat worldwide [1,2]. Annual cycles of stripe rust on winter wheat can be divided into four stages: oversummering, infection of seedlings in autumn, overwintering, and spring epidemic. As an obligate parasite, *Pst* has to survival under unfavorable weather in living host tissues [3,4], and therefore oversummering and overwintering are two bottlenecks for *Pst* epidemic development.

Temperature is an important factor influencing *Pst* development, including its survival during the winter [1,5–16]. The low limit of air temperature for *Pst* overwintering (November to February in China and North America) is believed to be around -6 to -7°C (monthly mean) [3,6,11,17–19] and could be as low as -10°C when wheat plants are under snow [3,5,17]. *Pst* development might halt when temperature is below -10°C [8]. Wheat-growing regions in China are divided into overwintering and non-overwintering regions for *Pst* based on winter temperatures [17,20]. Winter-hardiness of wheat cultivars and atmospheric moisture levels may also affect *Pst* overwintering [3,21]. Previous findings regarding the effect of environmental factors on *Pst* overwintering were based field observations, which reflect the overall effects of many external factors on *Pst* overwintering.

Studying *Pst* overwintering is hampered by the inability of assessing the amount of fungal biomass and its viability in wheat leaves efficiently. Recent development in PCR technology has enabled rapid detection and quantification of pathogens in plant tissues. The amount of *Pst* in leaves in early stages of infection prior to visual symptoms can be quantified using real-time quantitative PCR (qPCR) of DNA [22]. RNA is expected to be a better choice over DNA as an indicator of metabolic changes in response to external factors, hence indicating the viability. Quantification of RNA has recently been developed for plant pathogens including *Phytophthora parasitica* [23], *Pectobacterium atrosepticum* [24], *Puccinia triticina* [25–26], *P*. *striiformis* [25,27], *Verticillium dahliae* [28], and *V*. *longisporum* [28].

The current study was to 1) study the stability of DNA and RNA in *Pst* urediniospores stored under different conditions, 2) develop qPCR methods to quantify *Pst* DNA and RNA (cDNA) in plant tissues, and evaluate these two methods for quantifying *Pst* inside leaves at different stages of its development, 3) study *Pst* survival under several constant low temperatures using detached and attached leaves of one cultivar and 4) investigate the effect of cultivar winter-hardiness on *Pst* survival at low temperatures.

## Materials and Methods

### Fungal strains

A single *Pst* strain (CYR32—Chinese Yellow Rust 32, originally from Longnan, Gansu province, China) was used to develop qPCR methods. In addition, several other common wheat fungal pathogens were used to validate primer specificity, including *P*. *triticina* (from Hebei Agricultural University, China), *P*. *graminis* f. sp. *tritici* (from Shenyang Agricultural University, China), *Fusarium graminearum*, *Erysiphe graminis* f. sp. *tritici*, and *Bipolaris sorokiniana* (from Northwest A&F University, China).

### *Pst* multiplication and inoculation

CYR32 was multiplied in Taibai Observation Experimental Station, Shaanxi, China. Fresh urediniospores of CYR32 were inoculated onto seedlings of wheat cultivar Mingxian 169, a cultivar susceptible to all known *Pst* races. Seeds were sown in pots of 10-cm in diameter and 12-cm in depth, containing cow dung compost and soil (1:2, V/V). Ten days after sowing, seedlings with two leaves were inoculated with a fresh urediniospore suspension (50 mg urediniospoes/mL in deionized distilled water) of *Pst* CYR32 [29]. A fine paintbrush was used to daub spore suspension onto the adaxial surface of leaves until the whole leaf surface became wet without run-off [21,29]. The seedlings were kept in a dew chamber at 8–10°C in dark for 24 h immediately after inoculation and then grown on benches in a greenhouse at 14–17°C with a photoperiod of 10–14 h [29]. The plants in each pot were separated from plants in other pots with a transparent plastic cylinder to prevent contamination.

### DNA extraction

Wheat leaves (100 mg) were quickly frozen and transferred into a mortar pre-cooled with liquid nitrogen. Sixty microliters of Tris-HCl (pH 8.0) was added to the mortar and ground, and the resulting powder was transferred into a 1.5 mL eppendorf tube. Urediniospores were used as standards in qPCR; 10 mg urediniospores were put into a 1.5 mL eppendorf tube. Thirty microliters of Tris-HCl buffer (pH 8.0) was added into the tube. The tube was incubated for 5 min on ice to let Tris-HCl buffer permeate into urediniospores and was quickly frozen in liquid nitrogen. The urediniospores-Tris-HCl ice lump (S1 Fig) was transferred to a mortar pre-cooled with liquid nitrogen and ground, and the resulting powder was transferred into a new 1.5 mL centrifuge tube.

Once the powder was obtained, the following steps were used to extract DNA: 1) add 600 μL CTAB buffer (heated at 65°C before use) to the 1.5 mL eppendorf tube, vortex for 2 min and incubate for 40 min in a water bath at 65°C (invert the tube every 5 min); 2) incubate for 5 min on ice; 3) add 600 μL phenol: chloroform: isoamyl alcohol (25:24:1), and shake for 20 min; 4) centrifuge for 10 min at 15255× g at room temperature; 5) collect the aqueous phase and re-extract with 500 μL chloroform: isoamyl alcohol (24:1), and shake for 20 min; 6) repeat step 4; 7) collect the aqueous phase, add 50 μL of 3 M sodium acetate (pH 5.2) and 500 μL of isopropyl alcohol, and incubate for 30 min at -20°C; 8) centrifuge for 10 min at 15255× g at 4°C, wash the pellet with 75% ethanol and then with absolute ethanol, air-dry and dissolve it into 20 μL TE buffer (pH 8.0); 9) add 1.2 μL RNase (1 mg/mL), and incubate for 30 min at 37°C to remove RNA; and 10) measure DNA concentration using Nano Drop RND-2000 spectrophotometer (Thermo, USA), check DNA integrity through electrophoresis run in 1.0% agarose gel and visualize under AlphaImager System (CA, USA) after staining in an ethidium bromide solution (0.5 μg/mL) for 15 min.

### RNA extraction

Wheat leaves (100 mg) and urediniospores (10 mg) of *Pst* were transferred into a mortar and ground into powder separately as described for DNA extraction. The remaining extraction steps were the same for the powder of both wheat leaves and *Pst* urediniospores: 1) add 800 μL extraction buffer (0.02 M Tris-HCl pH 8.0, 1% SDS, 0.2 M NaCl, 5 mM EDTA), 10 μL of each 1% β-mercaptoethanol and 16% PVP, vortex the tube for 90 s 2–3 times, and incubate for 10 min on ice; 2) add 270 μL 5 M sodium acetate (pH 4.8) and 750 μL phenol: chloroform: isoamyl alcohol (25:24:1), mix well and incubate for 5 min on ice; 3) centrifuge for 10 min at 15255× g at 4°C, collect the aqueous phase, and re-extract with 700 μL chloroform and 300 μL of 5 M sodium acetate (pH 4.8); 4) precipitate RNA with 800 μL isopropyl alcohol and 100 μL of 3 M sodium acetate (pH 5.2) for 30 min at -20°C; 5) centrifuge for 10 min at 15255× g at 4°C, wash pellet with 75% ethanol and then with absolute ethanol, air-dry and dissolve it in 20 μL RNase free water; 6) add 5 μL 10× reaction buffer with MgCl_2_ for DNase I, 1 μL DNase I (RNasefree, 1 U/μL), 0.5 μL RNase inhibitor (40 U/μL), and ddH_2_O up to 50 μL, incubate for 30 min at 37°C; 7) precipitate with 150 μL isopropyl alcohol and 50 μL of 3 M sodium acetate (pH 5.2) for 30 min at -20°C; 8) repeat step 5; and 9) measure RNA concentration as described for DNA extraction.

### cDNA synthesis

cDNA was obtained through reverse transcription of RNA using PrimeScript RT Reagent Kit (Perfect Real Time) (TaKaRa, Japan). RNA was reversely transcribed at 37°C for 15 min, followed by heating at 85°C for 5 s. All steps followed the manufacturer’s instructions.

### PCR reaction

PCR reactions were performed in a thermal cycler (Bio-Rad MyCycler, USA) using a *Taq* (TakaRa, Japan). Each reaction contained 0.1 μL *Taq* (5 U/μL), 2.0 μL buffer (Mg^2+^ Free), 1.2 μL MgCl_2_ (25 mM), 1.6 μL dNTPs mixture (2.5 mM each), 0.4 μL primer mix (containing 20 μM of each primer), 1 μL (30 ng) of cDNA or DNA and distilled water up to 20 μL. The amplification conditions were as the following: a denaturation step at 94°C for 1 min followed by 35 amplification cycles consisting of denaturation at 94°C for 30 s, annealing at 60°C for 30 s and extension at 72°C for 30 s, with a final extension step at 72°C for 10 min. PCR products were separated in a 2.5% agarose gel and visualized under AlphaImager System (CA, USA) after staining in an ethidium bromide solution (0.5 μg/mL) for 35 min. *Pst* specific primer pair was designed from sequences of elongation factor 1 (*EF1*) (Forward primer: 5’-TTC GCC GTC CGT GAT ATG AGA CAA-3’; Reverse primer: 5’-ATG CGT ATC ATG GTG GTG GAG TGA-3’) [30]. Specificity of the primer pair against other common fungi on wheat leaves was also tested (S2A Fig). The size of PCR products using *Pst* cDNA and DNA as templates was 159 bp and 248 bp, respectively (S2B Fig). There were no other amplification products (S2C Fig).

### Real-time quantitative PCR

qPCR reactions were performed using an UltraSYBR Mixture kit (CWBIO, China). Each reaction contained 12.5 μL Ultra SYBR Mixture, 1.0 μL primer mix (containing 10 μM of each primer), 2 μL of cDNA or DNA (100 ng RNA and 140 ng DNA), and RNase free water up to 25 μL. The amplification conditions were as following: a denaturation step at 95°C for 3 min followed by 40 amplification cycles consisting of denaturation at 95°C for 30 s, annealing at 60°C for 30 s, and extension at 72°C for 30 s, with an amplification melting curve at 95°C for 1 min, 55°C for 1 min and followed by 81 cycles of 20 s from 55°C to 95°C with a step of 0.5°C. It was conducted on a real-time Thermal Cycler iQ5 (Bio-Rad, USA). The primer pair of *Pst EF1* was used. The iQ5 software 2.1 was used to estimate Cq value and PCR efficiency at the default settings. The mean Cq value and amplification efficiency (%) were calculated from three technical replicates for all individual samples.

### Constructing standard curves

For DNA standard curves, DNA was extracted from 10 mg of fresh *Pst* urediniospores and dissolved into 20 μL RNase and DNase free water. In the quantification system, 2 μL DNA (140 ng) was added, which was equivalent to the amount of DNA from 1.0×10^0^ mg (2 μL / 20 μL × 10 mg = 1.0 mg) urediniospores. The DNA standard was serially-diluted 10-fold using RNase and DNase free water, and with the final concentrations of 1.0×10^0^, 1.0×10^–1^, 1.0×10^–2^, 1.0×10^–3^, and 1.0×10^–4^ mg urediniospores. For RNA (cDNA) standard curves, RNA was extracted from 10 mg of fresh urediniospores and dissolved into 20 μL RNase free water, giving the RNA concentration of 400 ng/μL. Because of the constraint imposed by the transcription kit used in this study, only 2.5 μL RNA (1000 ng) can be reversely transcribed into 20 μL cDNA. In the quantification system, 2 μL cDNA was added, which was equivalent to the amount of cDNA from 1.25×10^–1^ mg (2.5 μL / 20 μL ×10 mg × 2 μL / 20 μL = 0.125 mg) urediniospores. The cDNA standard was serially diluted 10-folds using RNase free water to obtain concentrations of 1.25×10^–1^, 1.25×10^–2^, 1.25×10^–3^, 1.25×10^–4^, and 1.25×10^–5^ mg urediniospores. The standards were included in each quantification experiment. Quantified DNA or RNA (cDNA) was expressed in terms of *Pst* urediniospore weight.

### Stability of DNA and RNA in *Pst* urediniospores

DNA and RNA were quantified from three types (treatments) of CYR32 urediniospores over time: 1) dead urediniospores at ambient temperature, 2) fresh urediniospores at ambient temperature and 3) fresh urediniospores stored at -20°C. DNA and RNA were quantified at day 0, 4, 8, and 12. For each type of urediniospores, 5 mg of urediniospores were weighed and kept in a single tube. To kill the urediniospores, tubes with urediniospores were placed in a water bath at 60°C for 1 h. The effect of this water bath treatment on the viability of urediniospores was confirmed on 2.5% water agar (S3 Fig) as described previously [31]. On each of the four sampling events, DNA and RNA were extracted from urediniospores subjected to three treatments and measured using both spectrophotometer Nano-Drop RND-2000 (Thermo Scientific, USA) and qPCR. For each treatment at each sampling time, there were three replicates.

### Comparison of qPCR methods for quantifying *Pst* in inoculated leaves

Ten-day-old seedlings of cv. Mingxian 169 were inoculated with CYR32 urediniospores as described above. Leaf samples were collected 1 day before inoculation, and 1, 2, 3, 4, 5, 6, 7, 8, 9, 10, 11, 20, and 30 days post inoculation. On each sampling time point, six first-leaves were randomly collected. As the objective was to quantify *Pst* biomass inside leaves, surface urediniospores were washed with sterile distilled water three times before DNA or RNA extraction. Three of the six leaves were used for RNA extraction and the others for DNA extraction. Both DNA and RNA were quantified using qPCR as described above. The experiment was done three times.

### *Pst* survival in detached and attached leaves under low temperature

General experimental protocols are given in Table 1. Urediniospores were inoculated onto seedlings for use in all experiments, and the inoculation procedures were the same as described above. Initially, the survival of *Pst* in detached and attached leaves was studied only on Mingxian 169.

**Table 1:** Summary information on temperature, cultivar and sampling time in studying the survival of wheat stripe rust under constant temperatures.

Experiment	Sampling time (h) at different temperatures (°C)					Wheat cultivar
	0	-5	-10	-15	-20	
***Pst* survival in detached leaves**	0, 6, 12, 18, 24, 30, 36, 42, 48, 54	0, 6, 12, 18, 24, 30, 36, 42, 48, 54	0, 6, 12, 18, 24, 30, 36, 42, 48, 54	0, 6, 12, 18, 24, 30, 36, 42, 48, 54	0, 6, 12, 18, 24, 30, 36, 42, 48, 54	Mingxian 169
***Pst* survival in attached leaves**	0, 42, 48, 54	0, 42, 48, 54	0, 36, 42, 48	0, 18, 24, 30	0, 18, 24, 30	Mingxian 169
***Pst* survival in different wheat winter-hardiness**	0, 42, 48, 54	0, 42, 48, 54	0, 36, 42, 48	0, 12, 18, 24	0, 12, 18, 24	Mingxian 169, Xiaoyan 22, 01–314, Xinong 979, Lantian 15, Jingnong 411

For detached leaf assay, Mingxian 169 was grown in ten pots (10 × 12 cm per pot) with 15–20 seedlings per pot. Ten days after sowing, seedlings with two leaves were inoculated with suspension of fresh urediniospores as described above. When symptoms of stripe rust were first observed (ca. 12 days after inoculation) (S4 Fig), 30 leaves with symptoms were randomly collected from seedlings. Individual leaves were placed separately into 2.0 mL centrifuge tubes. These tubes were placed into a low-temperature-freezing cabinet THD-3010 (Tianheng, China), set to one of the five temperatures (0, -5, -10, -15, and -20°C). Three leaves (tubes) were randomly taken out from the cabinet for qPCR RNA assay at 0 (at the time of sampling), 6, 12, 18, 24, 30, 36, 42, 48 and 54 h at each temperature (Table 1). The experiment was done three times.

For attached leaf assay, the experiment was conducted in the same way as the detached leaf experiment except that seedlings with initial *Pst* symptoms were incubated in a low-temperature-freezing cabinet THD-3010 (Tianheng, China). Wheat leaves were cut off at four time points depending on the temperature; the exact sampling time was determined based on the results of the detached leaf assay (Table 1). This experiment was done three times.

To assess whether seedlings were still alive after incubation for 30 h at -15°C and -20°C, 48 h at -10°C, 54 h at 0°C and -5°C, the seedlings were moved to 4°C for 1 day, and then kept in a greenhouse (14–17°C with a photoperiod of 10–14 h) for one week before assessment for plant growth.

### Winter-hardiness evaluation of wheat cultivars

Seeds of wheat cultivars Mingxian 169, Xiaoyan 22, 01–314, Xinong 979, Lantian 15 and Jingnong 411 were sown in pots of 10-cm in diameter and 12-cm in depth, containing cow dung compost and soil (1:2, V/V). Twelve days after sowing, seedlings with three leaves were treated in low-temperature-freezing cabinet THD-3010 (Tianheng, China), set to one of the five temperatures (0, -5, -10, -15, and -20°C) for 36 h, respectively. The seedlings were kept at 4°C for 24 h after treatment and then moved to an artificial climate incubator with temperature of 18/23°C, photo period of 16/8 h, relative humidity of 60–80% and light intensity of 8000–10000 Lx for 15 d. The mortality of wheat leaves was calculated. The winter-hardiness was expressed using the temperature which causes 50% mortality of wheat leaves (LT50) [32].

### *Pst* survival in six cultivars under low temperatures

*Pst* survival under low temperatures was further studied on six wheat cultivars covering three levels of winter-hardiness: Mingxian 169 and Xiaoyan 22 (low winter-hardiness), Xinong 979 and 01–314 (moderate winter-hardiness) and Lantian 15 and Jingnong 411 (high winter-hardiness) (Table 2). Two pots of seedlings (15–20 seedlings per pot) with initial *Pst* symptoms of each cultivar were treated in the same way as described above for the attached leaf assay except that sampling times were 0, 12, 18 and 24 h at -15 and -20°C (Table 1). This experiment was done three times.

**Table 2:** Pedigrees, levels of winter-hardness, and breeding institute of wheat cultivars used to study the survival of wheat stripe rust under constant temperatures.

Cultivar name	Pedigree	Winter-hardiness*	Breeding Institute	Note
**Mingxian 169**	(S) LV-Shanxi	L	Northwest A&F University, Shaanxi, China	Landrace
**Xiaoyang 22**	(Xiaoyan 6 × 775-1) / Xiaoyan 107	L	Northwest A&F University, Shaanxi, China	Cultivar
**01-314**	Gaoyuan × {[ST142 × [(82N182 × 82Y11) × 47-2]] × [(78-17-3 × 83-228-3) × (Chanal × 338)] } F3	M	Institute of Geographic Sciences and Natural Resources Research, CAS, China	Cultivar
**Xinong 979**	Xinong 2611 / (918 × 95Xuan) F1	M	Northwest A&F University, Shaanxi, China	Cultivar
**Lantian 15**	Liantian10 / Ibis	H	Gansu Academy of Agricultural Sciences, Gansu, China	Cultivar
**Jingnong 411**	Fengkang 2 / Changfeng 1	H	Beijing Seed Company, Beijing, China	Cultivar

*L, low winter-hardiness; H, high winter-hardiness; M, moderate winter-hardiness.

### Data analysis

To compare quantification of *Pst* DNA and RNA in three types of urediniospores or in inoculated wheat leaves, a repeated measurement ANOVA was carried out in which the DNA and RNA were the main treatment factor. Repeated measurement ANOVA is necessary because of temporal correlation between assessments made over time on the same treatments. A post-hoc test for multiple treatment comparisons was carried with the Student-Newman-Keuls test using the pooled residual error from the ANOVA whenever significant treatment or interaction effects were revealed by ANOVA. For the comparisons on the inoculated wheat leaves, two separate ANOVAs were carried out: the first included only samples from 0 to 11 days after inoculation and the second included all time points. To reduce variance heterogeneity, data were logarithmically transformed. The replicate experiments were used as a block factor in ANOVA.

Viable *Pst* biomass in leaves was estimated as the amount of urediniospore based on qPCR of RNA (cDNA). The concentration of viable *Pst* in leaves was calculated as the ratio between quantified *Pst* RNA and leaf weight. The percentage of *Pst* (*PPS*) that survived a specific low temperature treatment at time *x* was calculated as:

$$PPS=\frac{A_{x}}{A_{0}}\times 100\%$$

where *A*_*0*_ and *A*_*x*_ are *Pst* concentrations in leaves at zero and *x* hour, respectively.

Repeated measurement ANOVA and the Student-Newman-Keuls test were used to assess the *Pst* survival on detached or attached leaves under different temperatures over time. To compare *Pst* survival on the detached and attached leaves, the leaf status (detached or attached) was treated as a factor in the repeated measurement ANOVA. In this analysis, only common sampling times between the attached and detached leaf experiments were included. Similarly, repeated measurement ANOVA and the Student-Newman-Keuls test were used to assess the effect of cultivar winter-hardiness and temperature on *Pst* survival on attached leaves over time. Three levels of winter hardiness were used in the ANOVA: low, moderate or high winter-hardiness. In all ANOVAs, replicate experiments were used as a block factor.

A key objective of the present study was to model the temporal dynamics of *Pst* survival at low temperatures, taking into account cultivar winter-hardiness. To study *Pst* survival over time on attached leaves at a given temperature, a two-parameter logistic model was fitted to each of 30 combinations of temperature and cultivar:

$$PPS=\frac{1}{1+\text{exp}\left(-r\left(t-m\right)\right)}$$

where *r* and *m* are the rate of *Pst* mortality and time to 50% mortality, respectively. Average *PPS* values over three replicate experiments were used to fit this logistic model to each combination of temperature and cultivar. This model assumes a 100% viable population at time zero. Finally, another logistic model was used to relate *r* or *m* to temperature in which winter-hardiness of cultivars was treated as a factor in fitting the logistic model.

All data analysis was carried out using software R (version 2.11.1).

## Results

### Stability of DNA and RNA in *Pst* urediniospores

**Fig 1:**
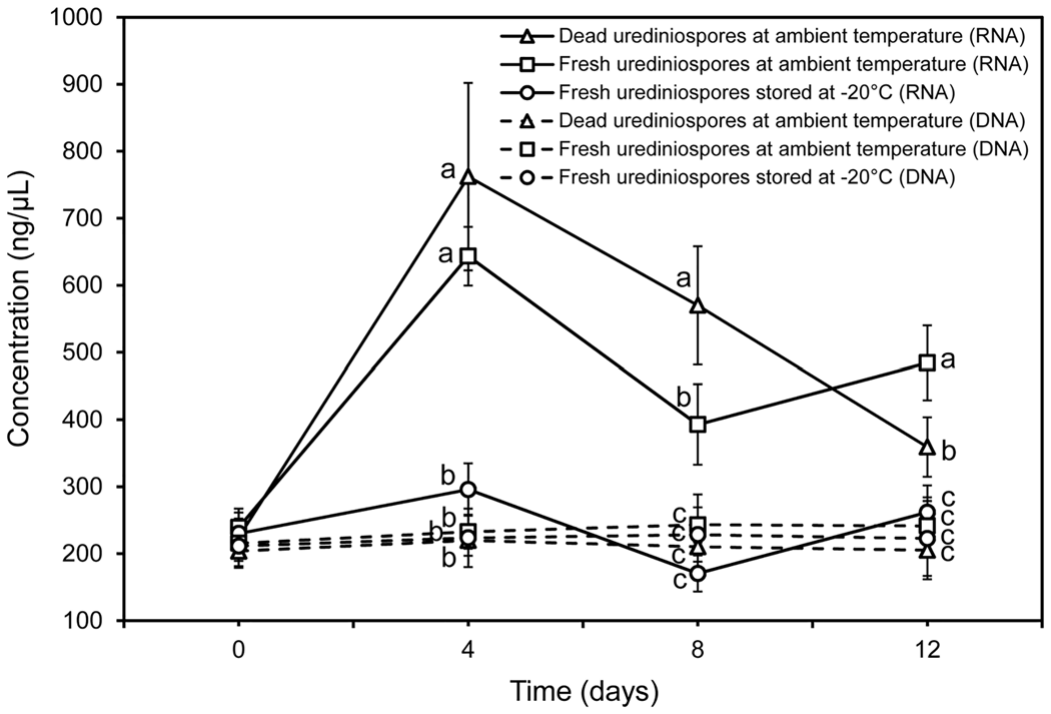
Estimated *Pst* DNA (dashed lines) and RNA (solid lines) from urediniospores subjected to three storage conditions over time. The concentration of DNA and RNA was measured using a spectrophotometer. Dashed lines with triangles, squares and circles represent DNA from dead urediniospores at ambient temperature, fresh urediniospores at ambient temperature and at -20°C, respectively. Solid lines with triangles, squares and circles represent RNA from dead urediniospores at ambient temperature, fresh urediniospores at ambient temperature and at -20°C, respectively. The experiment was done three times. The vertical bar for each treatment at each time point represents the standard deviation of the three mean values from three experiments; significant treatment differences were based on the pooled residual error in the repeated measurement ANOVA. The treatments with different letters on 4, 8, and 12 days means were significantly different at *P* = 0.05.

**Fig 2:**
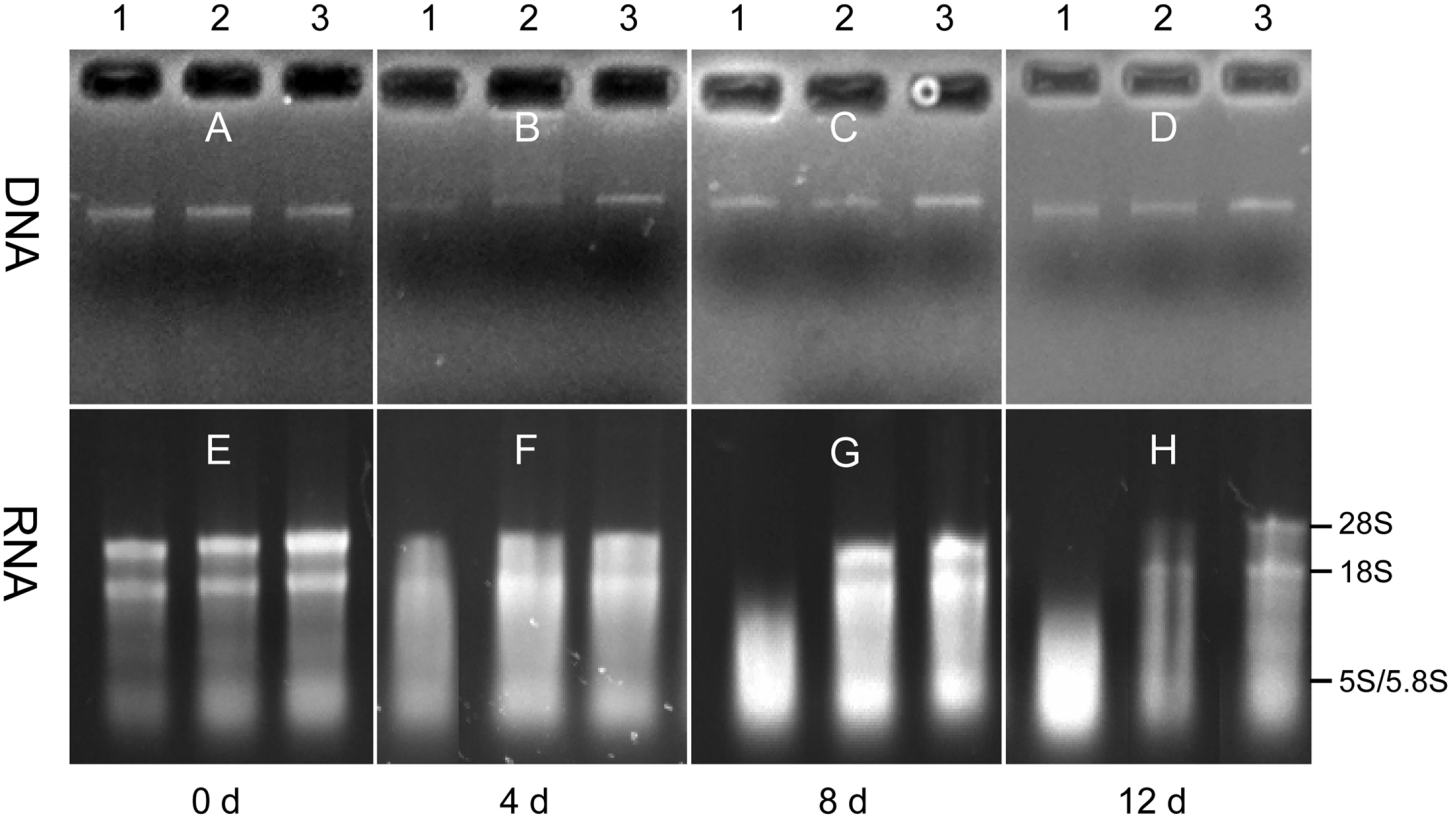
Integrity of DNA and RNA extracted from *Puccinia striiformis* f. sp. *tritici* (*Pst*) urediniospores stored at three conditions. Integrity of DNA (**A** to **D**) and RNA (**E** to **H**) was assessed using agarose gel (1.0%, W/V) on days 0, 4, 8 and 12. Lane 1, dead urediniospores (treated in a water bath at 60°C for 1 h) at ambient temperature; lane 2, fresh urediniospores at ambient temperature; and lane 3, fresh urediniospores at -20°C.

There were significant differences between DNA and RNA concentrations in three storage conditions (*P* < 0.001). The difference resulted primarily from large variation in RNA concentration over time (Fig 1). Compared with RNA, DNA concentration remained relatively stable over time (Fig 1). DNA integrity did not change much with time for all conditions (Fig 2A–2D). The 28S, 18S, 5.8S and 5S RNA were intact on day 0 (Fig 2E), but considerably degraded on day 4 for dead urediniospores (Fig 2F lane 1) with both 28S and 18S almost totally degraded on day 8 (Fig 2G lane 1). RNA extracted from viable urediniospores showed slight degradation on both day 4 and 8 (Fig 2F–2G lane 2). On day 12, RNA from viable urediniospores kept at ambient temperatures degraded more severely than that from urediniospores stored at -20°C (Fig 2H lane 2–3). The bands for 28S and 18S were clear for urediniospores stored at -20°C on all assessment days (Fig 2E–2H lane 3).

**Fig 3:**
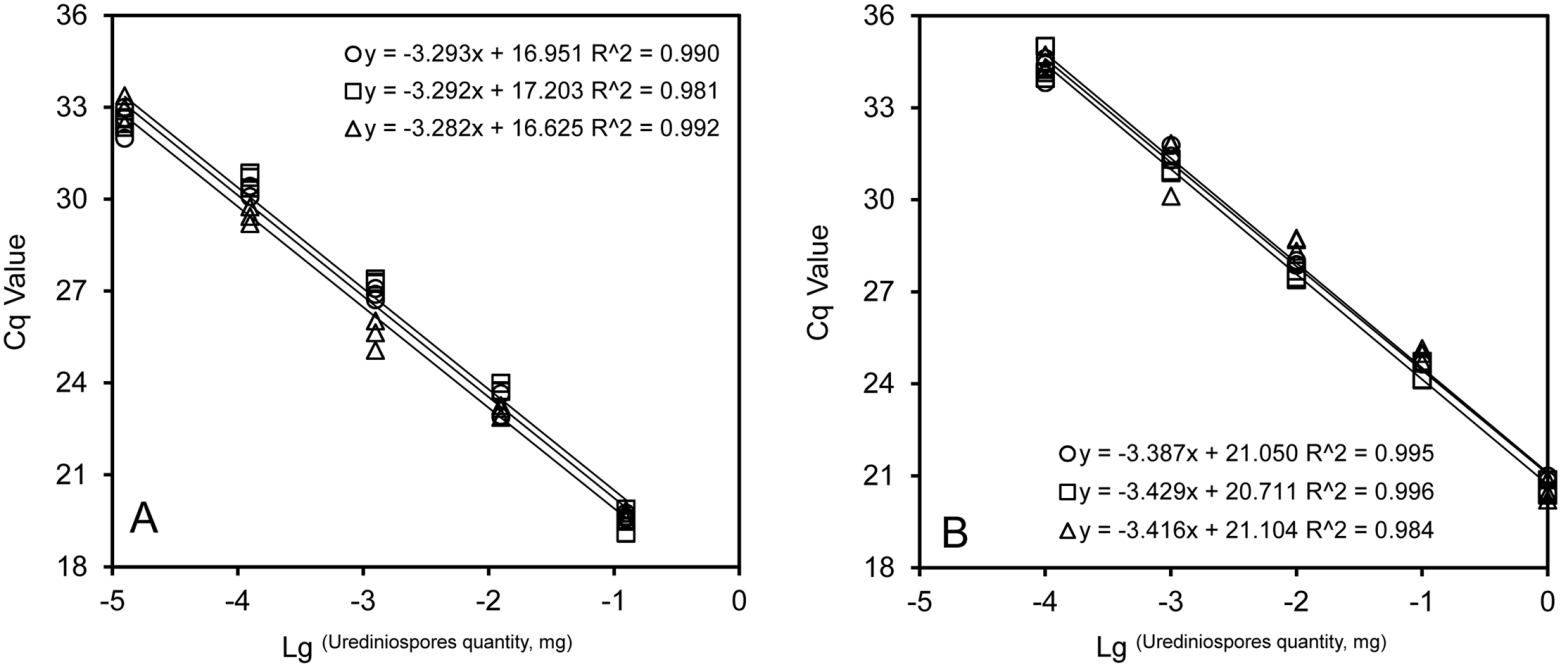
Example standard curves of real-time quantitative PCR of RNA (A) and DNA (B). The quantifications were expressed in terms of urediniospore weight. There were three replicates in each experiment.

**Fig 4:**
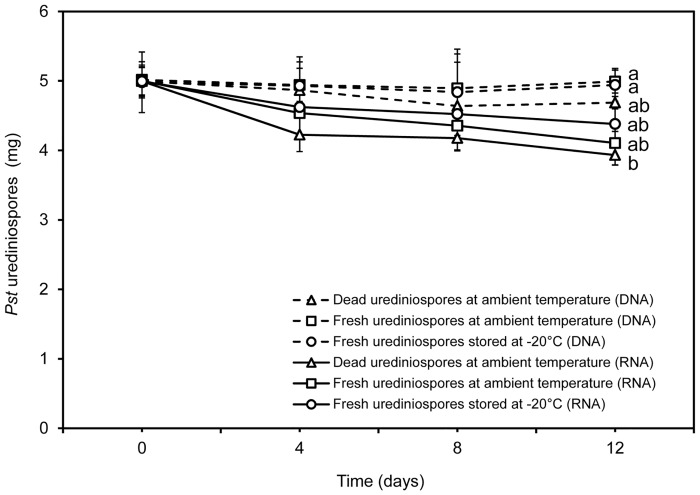
Estimated *Puccinia striiformis* f. sp. *tritici* of 5 mg urediniospores at three storage conditions using the qPCR of DNA (dashed lines) and RNA (solid lines). Dashed lines with triangles, squares and circles represent DNA from dead urediniospores at ambient temperature, fresh urediniospores at ambient temperature and at -20°C, respectively. Solid lines with triangles, squares and circles represent RNA from dead urediniospores at ambient temperature, and fresh urediniospores at ambient temperature and at -20°C, respectively. The experiment was done three times. The vertical bar of each treatment at each time point represents the standard deviation of the three mean values from three experiments; significant treatment differences were based on the pooled residual error in the repeated measurement ANOVA. Treatments with different letters for the 12-days means were significantly different at *P* = 0.05. There were no significant differences among the treatments on 0, 4 and 8 days at *P* = 0.05.

The amplified fragment size was 248 bp and 159 bp from DNA and cDNA of *Pst* using *EF1* primer pair, respectively (S2 Fig). Standard curves based on RNA (cDNA) and DNA were directly estimated using software iQ5 (Fig 3); the coefficient of determination (R^2^) for standard curves was greater than 0.98 in all quantification runs. Amplification efficiency ranged from 95.1% to 101.2% for RNA (cDNA) and from 97.3% to 99.7% for DNA. Five mg of *Pst* urediniospores under three storage conditions for 0–12 days were assayed using the qPCR of DNA and RNA (cDNA) (Fig 4). The amount of quantified *Pst* significantly differed (*P* < 0.01) between the two methods [qPCR of DNA and RNA (cDNA)]. However, both methods could not differentiate dead, fresh and dormant urediniospores except for a much lower amount of quantified *Pst* by qPCR of RNA for dead urediniospores at the ambient temperature (Fig 4).

### Quantification of *Pst* in leaves with qPCR of DNA and RNA

**Fig 5:**
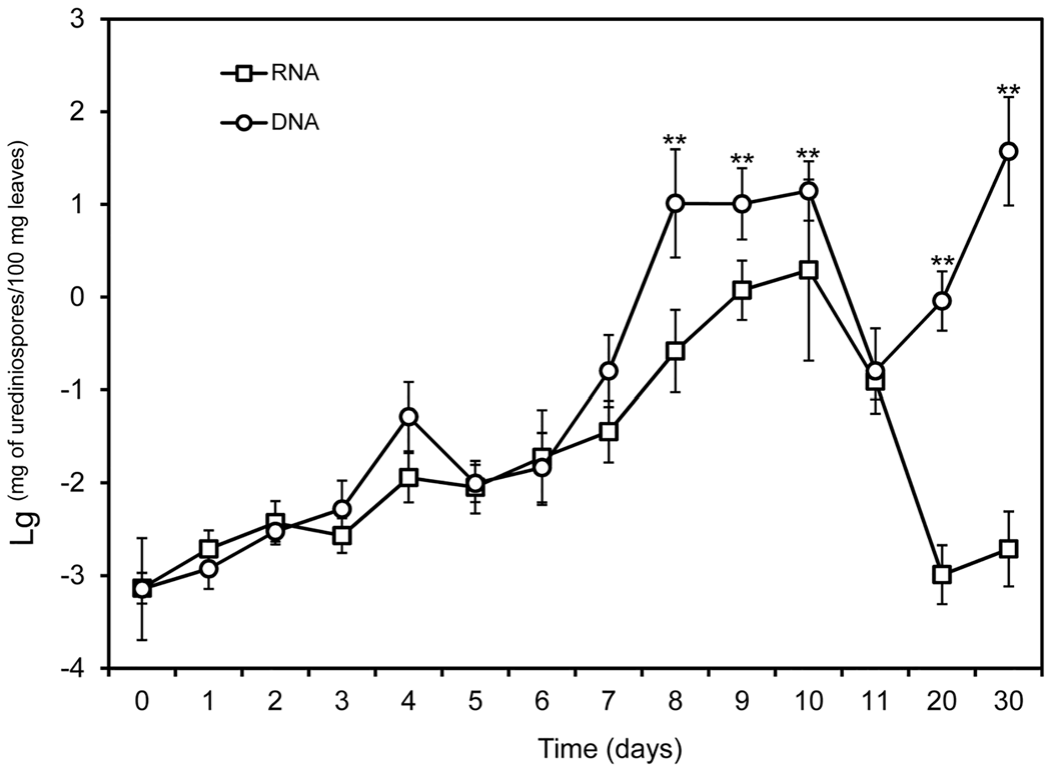
Quantified *Puccinia striiformis* f. sp. *tritici* in inoculated wheat leaves (cv. Mingxian 169) from day 0 to day 30 after inoculation using qPCR of DNA and RNA (cDNA). Urediniospores on the leaf surface were removed through washing leaves with distilled water. On day 20, more than 50% leaf area senesced, and on day 30, the leaves were completely senesced. The experiment was done three times. The vertical bar of a mean value represents the standard deviation among the three repeats of the experiment; significant treatment differences were based on the pooled residual error in the repeated measurement ANOVA. The level of quantified *Pst* DNA with “**” on 8, 9, 10, 20 and 30 days was significantly different from that based on the RNA quantification at *P* = 0.01. There were no significant (*P* > 0.05) differences between quantified levels of *Pst* DNA and RNA on other days.

Initially, quantified DNA and RNA followed a similar trend—increasing gradually over time and reaching a peak around day 10 (Fig 5). The overall level of DNA was greater (*P* < 0.05) than RNA from day 7 onwards. The greater increase in DNA from day 7 to day 10, relative to RNA, led to significant (*P* < 0.01) interactions between DNA-RNA differences and time (0–11 days). On day 20 and 30, RNA concentration decreased to nearly zero, in contrast to the sharp increase in DNA concentration (Fig 5). This increase in DNA and decrease in RNA on day 20 and 30 led to an even greater difference between DNA and RNA (*P* < 0.01) and also the interaction between DNA-RNA difference and time (*P* < 0.001) when all data were analyzed together. On day 20, more than 50% leaf area senesced (S5A Fig), and on day 30 the leaves were completely senesced (S5B Fig).

### *Pst* survival in detached and attached leaves under constant temperatures

**Fig 6:**
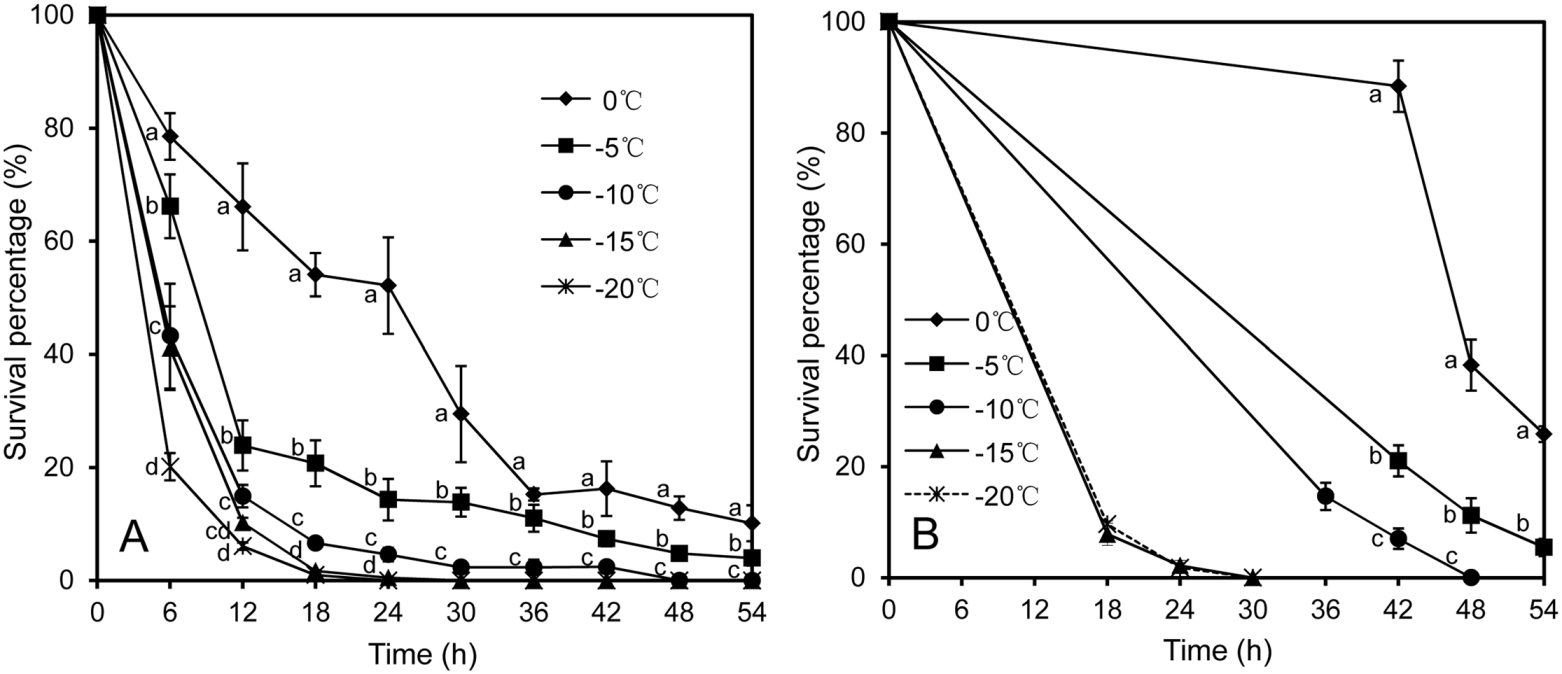
Percentage of viable *Puccinia striiformis* f. sp. *tritici* (*Pst*) quantified by qPCR of RNA in detached (A) and attached (B) leaves of cv. Mingxian 169 after incubation at different temperatures for a varying length of time. The experiment was done three times. The vertical bar of each mean value represents the standard deviation of the three mean values from the three experiments; significant treatment differences were based on the pooled residual error in the repeated measurement ANOVA. The temperature treatments with different letters at the same sampling time differed significantly at *P* = 0.05. There were no significant (*P* > 0.05) differences between -15°C and -20°C at the sampling time of 18, 24 and 30 h.

Overall, the percentage of *Pst* that survived in detached and attached leaves decreased (*P* < 0.001) over time at all test temperatures (Fig 6A–6B). *Pst* survival differed greatly (*P* < 0.001) between attached and detached leaves. A greater (*P* < 0.001) percentage of *Pst* survived in attached leaves than in detached leaves after prolonged incubation: at 0°C during 42–54 h, at -5°C during 42–48 h, at -10°C during 36–42 h and at -15°C and -20°C after 18 h. This difference was most pronounced at 0°C (Fig 6B). Viable *Pst* was still detected after incubation at 0°C and -5°C for 54 h in both detached and attached leaves. The percentage of *Pst* that survived was 10.1% and 4.0% at 0°C and -5°C, respectively after incubation for 54 h in detached leaves; the corresponding values in attached leaves were 25.8% and 5.5%, respectively. The amount of viable *Pst* was below the limit of detection after incubation at -10°C for 48 h, at -15°C for 30 h in both attached and detached leaves and at -20°C for 24 h in detached leaves and for 30 h in attached leaves. Plants that were incubated at -20°C for 30 h, -15°C for 30 h, -10°C for 42 h, -5°C and 0°C for 54 h were still alive when transferred into 4°C for 1 d and then maintained under normal conditions in a greenhouse for one week.

### Winter-hardiness of six cultivars

The wheat leaf mortality of six cultivars increased as temperature decreased. The relationship between wheat leaf mortality and temperature of Mingxian 169, Xiaoyan 22, 01–314, Xinong 979, Lantian 15 and Jingnong 411 was well described by a logistic model, and classified as three types: type I- Mingxian 169 and Xiaoyan 22, type II-01-314 and Xinong 979 and type III- Lantian 15 and Jingnong 411 (S6 Fig). The LT50 of the three types was -6.5°C, -8.3°C and -11.8°C, respectively. Mingxian 169 and Xiaoyan 22 were low winter-hardiness cultivars, Xinong 979 and 01–314 were moderate winter-hardiness cultivars and Lantian 15 and Jingnong 411 were high winter-hardiness cultivars (Table 2).

### *Pst* survival in six cultivars under low temperatures

**Fig 7:**
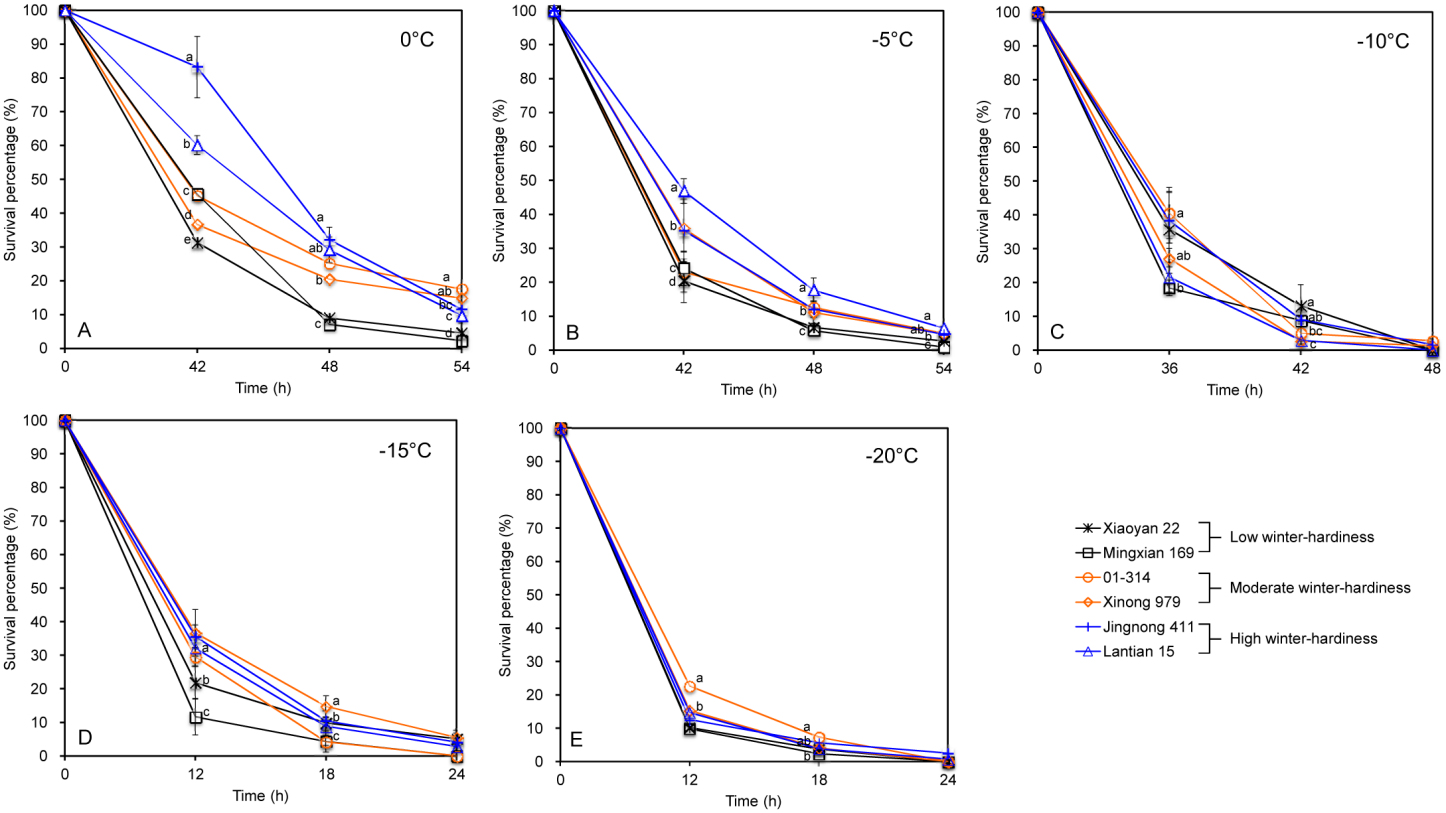
Percentage of viable *Puccinia striiformis* f. sp. *tritici* (*Pst*) quantified by qPCR of RNA in attached leaves of six cultivars with different levels of winter-hardiness. A: 0°C; B: -5°C; C: 10°C; D: -15°C; and E: -20°C. The experiment was conducted three times. The vertical bar of each mean value represents the standard deviation of the three mean values of the three experiments; significant treatment differences were based on the pooled residual error in the repeated measurement ANOVA. The winter-hardiness cultivar treatments with different letters at the same sampling time differed significantly at *P* = 0.05.

At 0°C and -5°C, the percentage of viable *Pst* increased with increasing cultivar winter-hardiness (Fig 7A–7B). However, such differences between cultivars were very small at other test temperatures (Fig 7C–7E).

**Fig 8:**
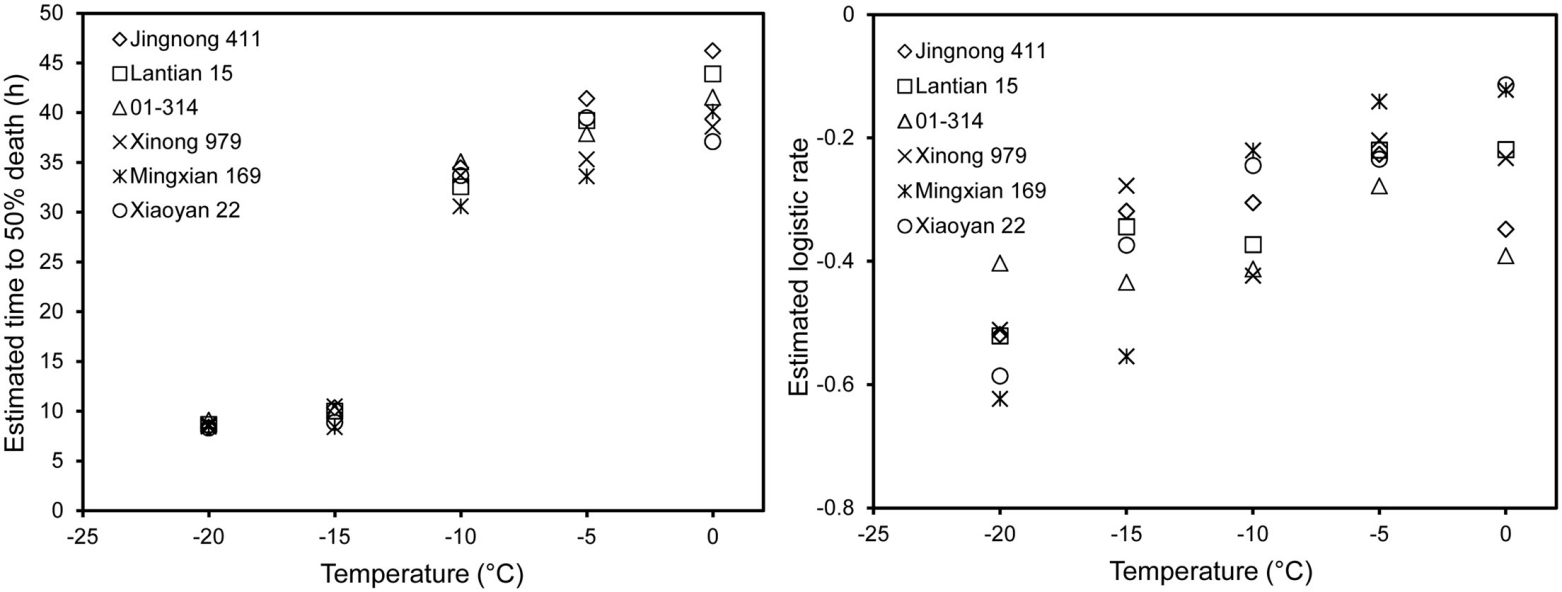
Determination of two parameters, time to 50% mortality (A) and the logistic rate (B), in the logistic models describing the dynamics of viable *Puccinia striiformis* f. sp. *tritici* over time for wheat cultivars with different levels of winter hardness tested at various low temperatures.

The temporal dynamics of viable *Pst* at a given temperature was well described by a logistic model with two parameters: rate of *Pst* mortality (*r*) and time to 50% mortality (*m*). This model explained more than 99% of the total variability in average viable *Pst* over time for all 30 datasets (five temperatures × six cultivars). Both *r* and *m* were related to temperature though the relationship of *m* with temperature (Fig 8A) was stronger than that of *r* with temperature (Fig 8B). Overall, *m* decreased with decreasing temperature; differences in *m* among cultivars were primarily at 0°C and -5°C. In contrast, there were large differences in *r* across all test temperatures; however, there appeared to be no discernable pattern of *r* in relation to either of cultivar or winter hardiness.

The relationship between *m* and temperature was well described by a logistic model with four parameters (Fig 8A):

$$m=A+\frac{C}{1+\text{exp}\left(-r_{1}\left(t-m_{1}\right)\right)}$$

For all six cultivars, *C* = 31.1, *r*_*1*_ = 0.90 and *m*_*1*_ = -11.53; *A* = 9.86, 8.39 and 7.21 for cultivars with high, moderate and low winter-hardiness, respectively. In this model, parameters *A* and (*A*+*C*) are the respective minimum and maximum of *m* (time to 50% mortality). This model accounted for 97.7% of the total variability in *m*.

## Discussion

This is the first published study that quantifies viable *Pst* in wheat leaves using qPCR of RNA. RNA is usually more easily degraded than DNA. Quantified DNA concentration based on qPCR may remain stable for longer than the corresponding estimated RNA. However, whether qPCR quantification of RNA is better than the corresponding qPCR of DNA as an indicator of viable fungal structure depends on the type of fungal structure. For urediniospores, both qPCR of RNA and DNA cannot distinguish dormant from dead spores. In contrast, qPCR of RNA, but not DNA, can determine whether hyphae inside leaves are viable. Two possible reasons may explain why qPCR of RNA cannot distinguish dead urediniospores from viable ones. First, RNA is closely linked to protein synthesis (hence concentration), which varies considerably according to the organism’s metabolic needs [33]. In the present study, viable spores were not maintained under conditions that are conducive to spore germination since the objective was to assess the detection of viable (yet non-germinating spores) using qPCR methods. The overall level of metabolism in non-germinating spores may be below the detection level of the present qPCR method. Second, the present qPCR method only quantifies mRNA for a particular gene—elongation factor 1, which relates to the production of cytoskeleton [34–36]. In non-germinating spores, metabolic activity related to cytoskeleton production may be very low and cannot be detected by the present qPCR method. Further research is needed to determine whether viable non-germinating spores could be distinguished from dead ones based on qPCR quantification of other genes.

There were no significant differences between the two qPCR methods (RNA and DNA) in the amount of quantified *Pst* in inoculated wheat leaves from 0 to 11 days after inoculation. However, qPCR of DNA resulted in a much greater estimate of *Pst* than qPCR of RNA post sporulation (12 to 30 days). Usually, DNA can remain intact for a longer time after cell death than RNA; therefore, qPCR of DNA may overestimate the amount of *Pst* because of dead urediniospores or mycelia. Thus, both qPCR of RNA and DNA can be used to estimate *Pst* amount during the initial latent period but only qPCR of RNA should be used post sporulation to estimate viable *Pst*.

The percentage of *Pst* that is viable in detached and attached leaves decreased over time under low temperatures. Low temperature is believed to reduce pathogen survival in infected leaves [1]. As expected, *Pst* survival is greatly reduced when temperature is below -10°C. However, some reports claimed that *Pst* can survive as long as the infected foliage remains green during winter [3–4]. The present result indicated that *Pst* in attached green leaves did not survive at -20°C after 30 h, however the plants were still alive at this condition. Saulescu and Braun [37] also reported that plants may remain alive even up to 48 h at -18 to -20°C. Under the condition of this study, we conclude that *Pst* mycelia inside green leaves can be killed by low temperature and that the decrease in viable *Pst* is not all due to death of leaf tissues. It should be noted that seedlings were transferred to low temperatures without adequate acclimatization time in the present study. Wheat plants that have grown to the tillering stage and been slowly acclimated to low temperatures may survive better, which may also influence *Pst* survival. Thus, it would be interesting to study whether wheat plant age and acclimatization time could affect *Pst* survival.

Winter-hardiness of wheat cultivars may affect *Pst* overwintering [3,21]. The present results suggested that the percentage of viable *Pst* was greater on cultivars with strong winter-hardiness than those with weak winter-hardiness mainly at 0°C and -5°C. Under the conditions of this study, both temperature and cultivar winter-hardiness affect *Pst* survival in winter, but temperature has more impact on determining the *Pst* survival.

Many models have been developed to describe the relationship between winter temperature and stripe rust epidemics based on field observations [6,7,11,38] and were further revised [16] to predict the spring epidemic. For the first time, we have developed models relating *Pst* mortality to temperature and cultivar winter-hardiness. These models predict the shortest and longest *Pst* survival on cultivars of low and high winter-hardiness, respectively, at a particular temperature. It should be noted that we assumed that there is no mortality when temperature > 0°C which was shown previously [5].

In summary, we have developed a method based on qPCR of RNA to quantify viable *Pst* biomass in leaf tissues. This qPCR-RNA method can be used in detecting and quantifying the pathogen prior to symptoms, which may be used as an input for disease prediction. A mathematical model was derived to relate *Pst* survival to temperature and cultivar winter-hardiness, which can be used predict *Pst* survival under fluctuating conditions. However, further data on field *Pst* survival are needed to assess whether inclusion of other factors (e.g. moisture and snow cover) could improve model predictions of *Pst* survival over the winter. For this purpose, we are sampling wheat leaves in early spring from plants showing symptoms in the autumn at many locations to quantify viable *Pst* biomass. These data will be used to evaluate and, if necessary, improve this temperature-only model. Availability of such a predictive model will be valuable to assist growers in timing fungicide applications in given geographical regions.

## Supporting Information

S1 Fig Urediniospores of *Puccinia striiformis* f. sp. *tritici* were mixed with Tris-HCl buffer (pH 8.0) and then quickly frozen in liquid nitrogen, becoming a urediniospores-Tris-HCl ice lump in order to reduce loss of *Pst* urediniospores. (TIFF)

S2 Fig Specificity of primer pair *EF1* of *Puccinia striiformis* f. sp. *tritici* (*Pst*). A: the primer pair *EF1* distinguished *Pst* from other pathogens. Lane M, DNA ladder MD 101 (TianGen, China); lane 1, negative control using sterile distilled water; lane 2, negative control using cDNA of healthy wheat leaves; lane 3 to 7, cDNA from *Pst*, *P*. *triticina*, *P*. *graminis* f. sp. *tritici*, *Fusarium graminearum*, and *Blumeria graminis* f. sp. *tritici*, respectively. B: cDNA (159 bp) and DNA (248 bp) band amplified using *EF1* primer from cDNA and DNA of *Pst*, respectively. Lane M, DNA ladder MD 101 (TianGen, China); lane 1, negative control using sterile distilled water; lane 2 to 3, cDNA and DNA of *Pst* urediniospores, respectively. C: Melting curve of real-time quantitative PCR amplification using primer pair *EF1* of *Pst*.
(TIFF)

S3 Fig Germination of urediniospores of *Puccinia striiformis* f. sp. *tritici* isolate CYR32. (A) fresh urediniospores; (B) fresh urediniospores spread onto 2.5% water agar surface and incubated at 12°C in dark with relative humidity 85% for 12 h; (C) fresh urediniospore heated in a water bath at 60°C for 1 h; (D) fresh urediniospore heated in a water bath at 60°C for 1 h and spread onto 2.5% water agar surface and incubated at 12°C in the dark with relative humidity 85% for 12 h.
(TIFF)

S4 Fig Initial symptoms of wheat stripe rust caused by *Puccinia striiformis* f. sp. *tritici* on inoculated leaves. (TIFF)

S5 Fig Stripe rust symptoms on leaves and leaf status of wheat cv. Mingxian 169 20 days (A) and 30 (B) days after inoculation. (TIFF)

S6 FigThe relationship between wheat leaf mortality (*LM*) and temperature (*T*).The relationship between wheat leaf mortality (*LM*) and temperature (*T*) of Mingxian 169 and Xiaoyan 22, 01–314 and Xinong 979 and Lantian 15 and Jingnong 411 were well described by the following logistic models:
$$\begin{array}{l} LM_{Mingxian169-Xiaoyan22}=\frac{1}{1+e^{3.687+0.568T}},R^{2}=0.809\\ LM_{01-314-Xinong979}=\frac{1}{1+e^{2.500+0.301T}},R^{2}=0.813\\ LM_{Lantian15-Jingnong411}=\frac{1}{1+e^{3.161+0.269T}},R^{2}=0.853 \end{array}$$
The vertical bar of each mean value represents the standard deviation of the three mean values of the three experiments; significant treatment differences were based on the pooled residual error in the repeated measurement ANOVA. The group of cultivar with similar winter-hardiness with different letters at the same temperature differed significantly at *P* = 0.05.(TIFF)
