# Supplementary figures and images for: Effect of Low Temperature and Wheat Winter-Hardiness on Survival of Puccinia striiformis f. sp. tritici under Controlled Conditions
Source: PLoS One. 2015 Jun 17;10(6):e0130691. doi: 10.1371/journal.pone.0130691 (PMC4470655; doi:10.1371/journal.pone.0130691)

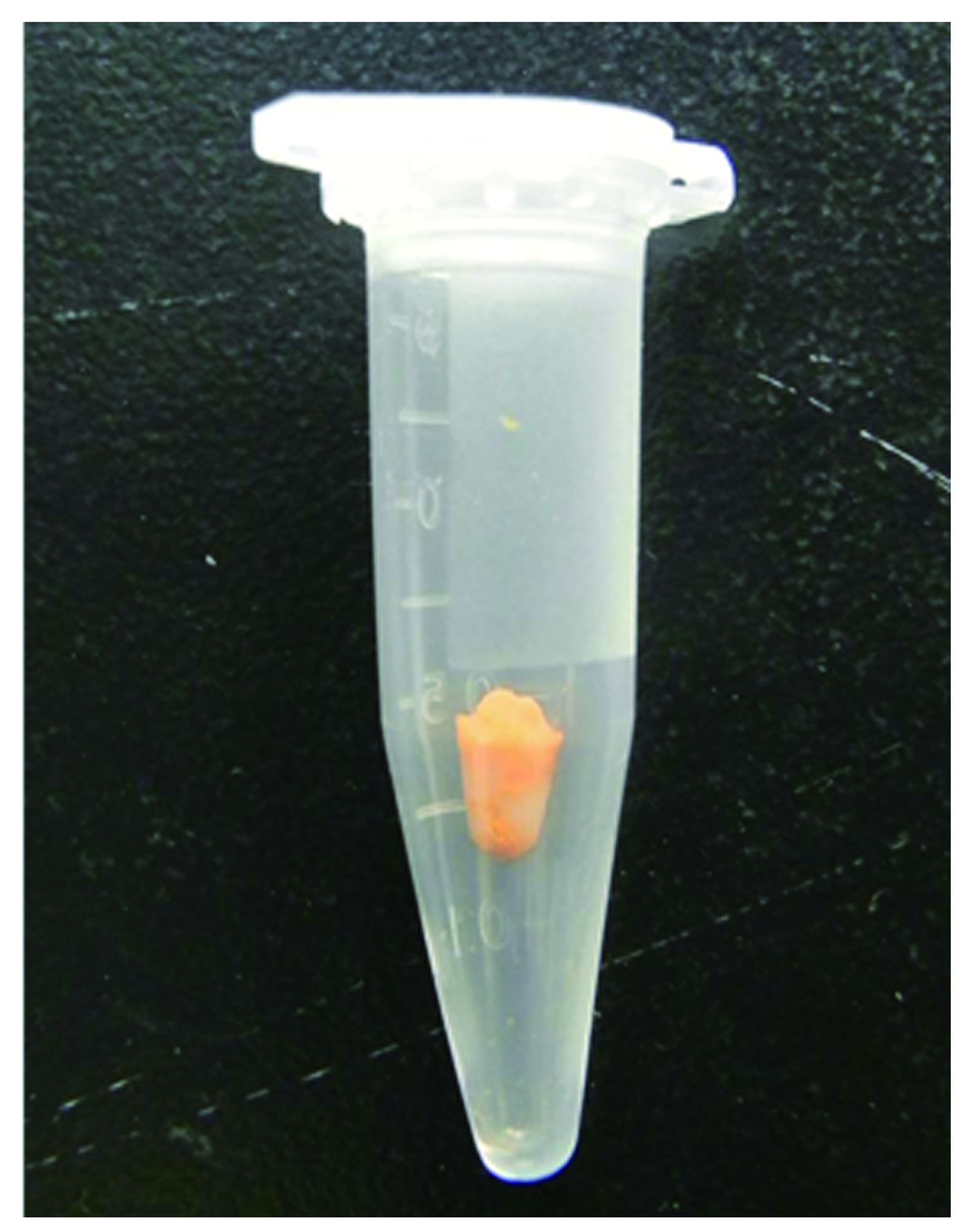

Supplement: S1 Fig — (TIFF) [file pone.0130691.s001.tiff]

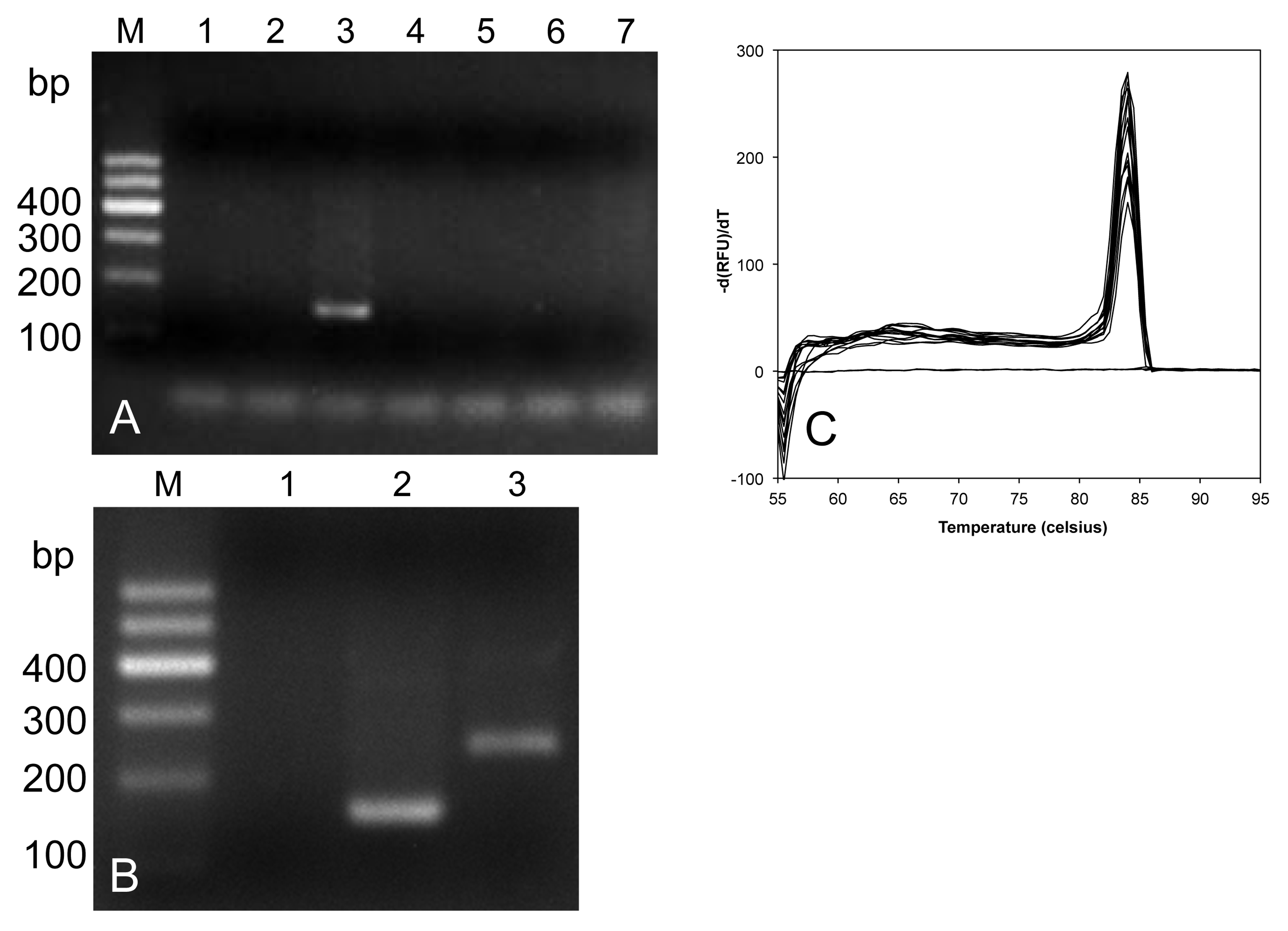

Supplement: S2 Fig — A: the primer pair EF1 distinguished Pst from other pathogens. Lane M, DNA ladder MD 101 (TianGen, China); lane 1, negative control using sterile distilled water; lane 2, negative control using cDNA of healthy wheat leaves; lane 3 to 7, cDNA from Pst, P. triticina, P. graminis f. sp. tritici, Fusarium graminearum, and Blumeria graminis f. sp. tritici, respectively. B: cDNA (159 bp) and DNA (248 bp) band amplified using EF1 primer from cDNA and DNA of Pst, respectively. Lane M, DNA ladder MD 101 (TianGen, China); lane 1, negative control using sterile distilled water; lane 2 to 3, cDNA and DNA of Pst urediniospores, respectively. C: Melting curve of real-time quantitative PCR amplification using primer pair EF1 of Pst. (TIFF) [file pone.0130691.s002.tiff]

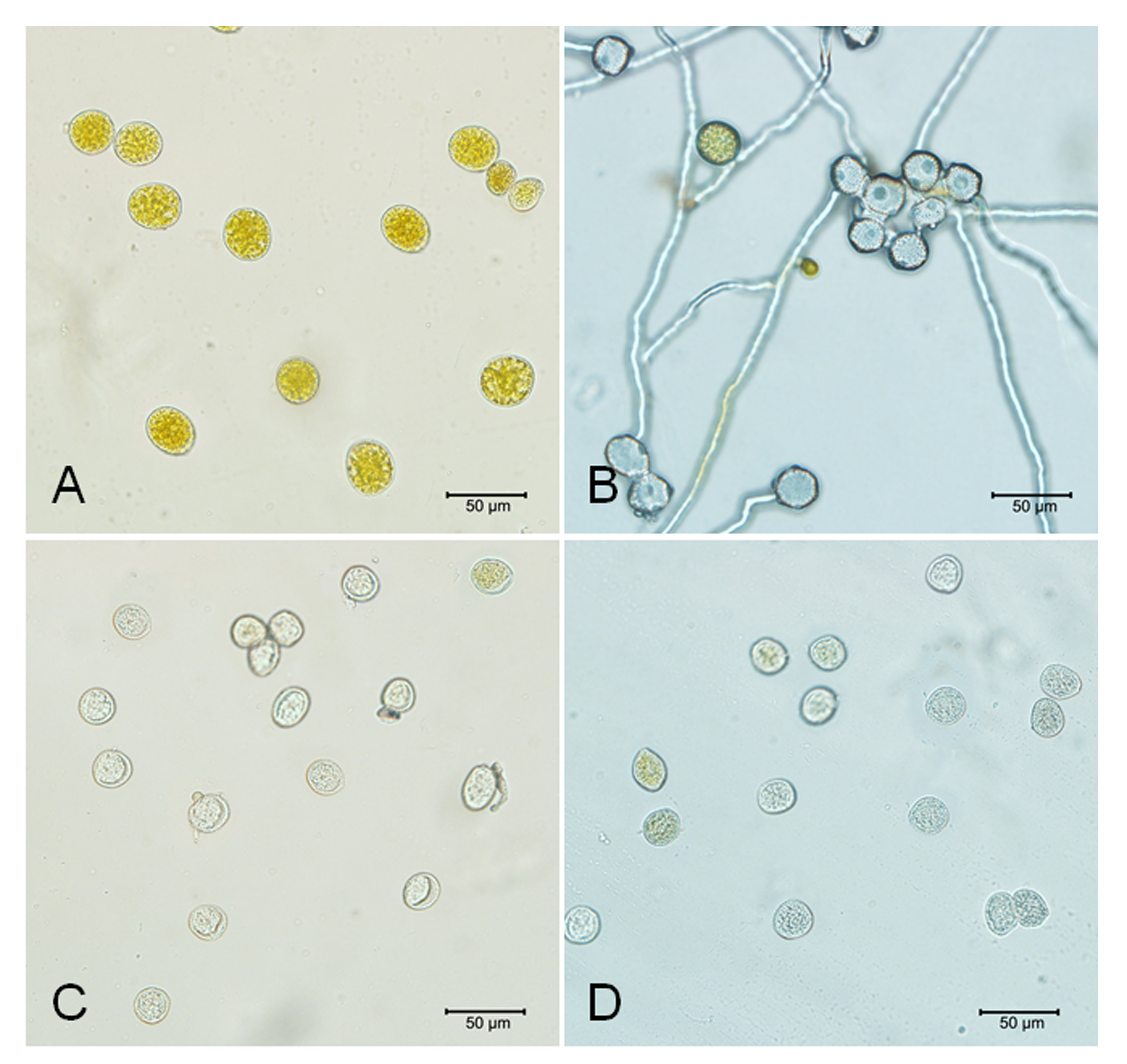

Supplement: S3 Fig — (A) fresh urediniospores; (B) fresh urediniospores spread onto 2.5% water agar surface and incubated at 12°C in dark with relative humidity 85% for 12 h; (C) fresh urediniospore heated in a water bath at 60°C for 1 h; (D) fresh urediniospore heated in a water bath at 60°C for 1 h and spread onto 2.5% water agar surface and incubated at 12°C in the dark with relative humidity 85% for 12 h. (TIFF) [file pone.0130691.s003.tiff]

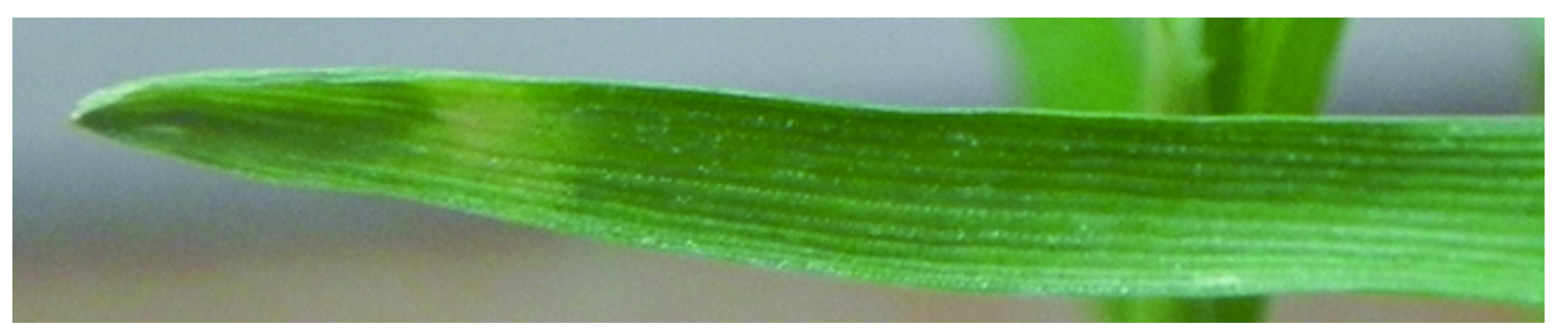

Supplement: S4 Fig — (TIFF) [file pone.0130691.s004.tiff]

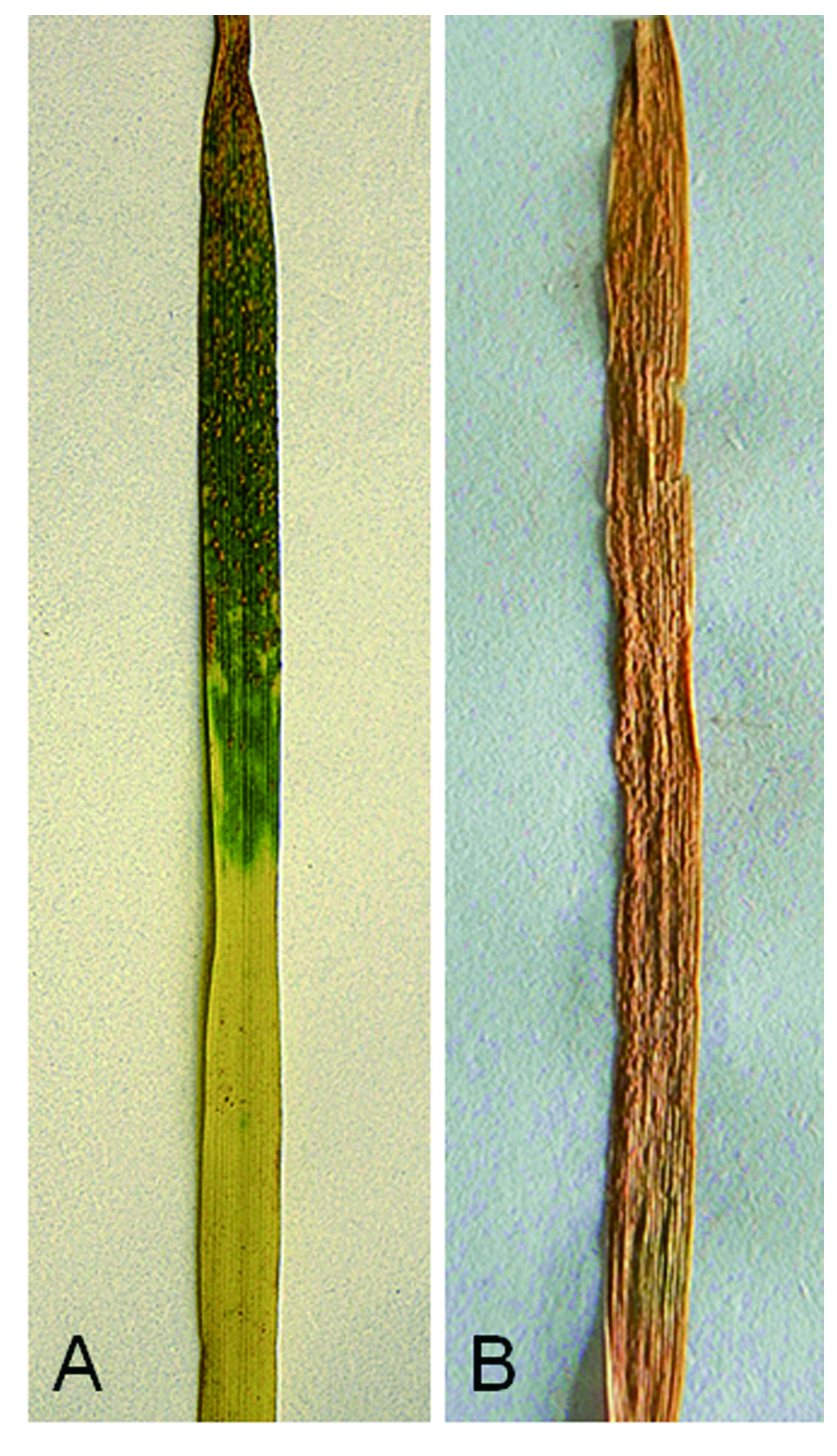

Supplement: S5 Fig — (TIFF) [file pone.0130691.s005.tiff]

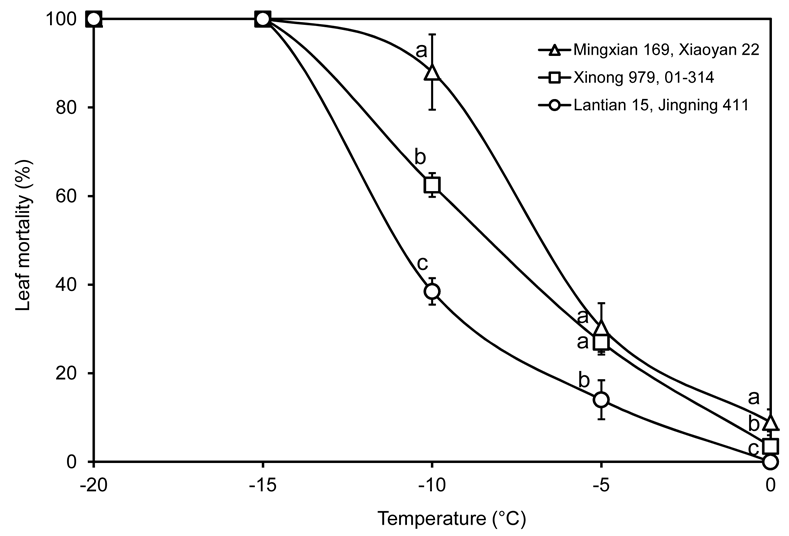

Supplement: S6 Fig — The relationship between wheat leaf mortality (LM) and temperature (T) of Mingxian 169 and Xiaoyan 22, 01–314 and Xinong 979 and Lantian 15 and Jingnong 411 were well described by the following logistic models: LMMingxian169−Xiaoyan22=11+e3.687+0.568T,R2=0.809LM01−314−Xinong979=11+e2.500+0.301T,R2=0.813LMLantian15−Jingnong411=11+e3.161+0.269T,R2=0.853 The vertical bar of each mean value represents the standard deviation of the three mean values of the three experiments; significant treatment differences were based on the pooled residual error in the repeated measurement ANOVA. The group of cultivar with similar winter-hardiness with different letters at the same temperature differed significantly at P = 0.05. (TIFF) [file pone.0130691.s006.tiff]
